# On the Effects of Menstruation on the Milk of Nurses

**Published:** 1853-11

**Authors:** 


*On the Effects of Menstruation on the Milk of Nurses.* By MM. BECQUEREL & VERNOIS.—Upon this effect, which the occurrence of menstruation exerts in women who are suckling, there is discrepancy of opinion among authors, the majority, however, with the public at large, believing in its deteriorating influence. So great is the difficulty in obtaining true statements upon this point, that among the great number of hired nurses in Paris, the authors have only been able to examine the condition of the milk in three women while actually menstruating. In these the density of the fluid was found slightly diminished, as was the proportion of sugar, and the proportion of water was sensibly so. The solid parts were notably increased, especially the caseine. The authors cannot believe that such changes in composition can induce any mischief beyond some temporary derangement in the digestive organs, and even this might be prevented by causing the child to suck less, and letting it drink a little sugared water, to replace the sugar and water lost during menstruation.

In the discussion that followed reading the paper, M. Roger observed, that while attached to the Office for Nurses, he had paid considerable attention to this point, and that he had arrived at the following conclusions:—If the menses reappear easily, without pain or derangement of the nurse's health, while her milk is under 12 or 15 months old, and the quantity of blood lost is normal and moderate, the quantity of milk does not become diminished, or its qualities altered, and the child does not suffer from its use. If, however, the menses are too abundant or too frequent, the milk may diminish in quantity or disappear. The same effect is also produced, though more slowly, in some days or weeks, when the menses are prolonged for a week, so that the loss is considerable. The milk will much more certainly dry up if the menses reappear at an advanced period of lactation—this being then the signal of the imperfection and approaching termination of the secretion.

When the milk becomes thus diminished, it rarely exhibits the physical characters of the poor milk; but by its density, whiteness, and the excess in number and size of its globules, it more approaches in character and richness cow's milk. When the menstrual epochs reappear with difficulty, and are attended with pain, indigestion, diarrhœa, &c., or are preceded or followed by leucorrhœa, the child may suffer symptoms due to indigestion induced by the altered characters of the milk—the alteration of the milk chiefly consisting in increase in the number and size of the globules. These influences are, however, only temporary, and the milk soon recovers its normal character. The ailments which the child hence suffers are only temporary, and have been greatly exaggerated. —*Ibid, from L' Union Medicale*, No. 70.

